# Ant Colonies Prefer Infected over Uninfected Nest Sites

**DOI:** 10.1371/journal.pone.0111961

**Published:** 2014-11-05

**Authors:** Luigi Pontieri, Svjetlana Vojvodic, Riley Graham, Jes Søe Pedersen, Timothy A. Linksvayer

**Affiliations:** 1 Centre for Social Evolution, Department of Biology, University of Copenhagen, Copenhagen, Denmark; 2 Center for Insect Science, Department of Ecology and Evolutionary Biology, University of Arizona, Tucson, Arizona, United States of America; 3 Department of Biological Sciences, Rowan University, Glassboro, New Jersey, United States of America; 4 Department of Biology, University of Pennsylvania, Philadelphia, Pennsylvania, United States of America; University of Sheffield, United Kingdom

## Abstract

During colony relocation, the selection of a new nest involves exploration and assessment of potential sites followed by colony movement on the basis of a collective decision making process. Hygiene and pathogen load of the potential nest sites are factors worker scouts might evaluate, given the high risk of epidemics in group-living animals. Choosing nest sites free of pathogens is hypothesized to be highly efficient in invasive ants as each of their introduced populations is often an open network of nests exchanging individuals (unicolonial) with frequent relocation into new nest sites and low genetic diversity, likely making these species particularly vulnerable to parasites and diseases. We investigated the nest site preference of the invasive pharaoh ant, *Monomorium pharaonis*, through binary choice tests between three nest types: nests containing dead nestmates overgrown with sporulating mycelium of the entomopathogenic fungus *Metarhizium brunneum* (infected nests), nests containing nestmates killed by freezing (uninfected nests), and empty nests. In contrast to the expectation pharaoh ant colonies preferentially (84%) moved into the infected nest when presented with the choice of an infected and an uninfected nest. The ants had an intermediate preference for empty nests. Pharaoh ants display an overall preference for infected nests during colony relocation. While we cannot rule out that the ants are actually manipulated by the pathogen, we propose that this preference might be an adaptive strategy by the host to “immunize” the colony against future exposure to the same pathogenic fungus.

## Introduction

In social insects, the selection of a suitable nest site during colony establishment and relocation is critical for colony success. Resource availability, intra- and inter-specific competition level, as well as abiotic factors characterizing the new site can strongly affect colony fitness [1]. The selection of a new nest site is particularly critical during colony relocation, a common phenomenon in eusocial insects [2], as migrating between nests is risky and energetically costly, and the colony usually has to select the best site among several candidates. An accurate and effective assessment of the properties of the potential nest sites is thus crucial. Studies conducted in honeybees [3] and *Temnothorax* ants [4] have shown that individual scouts explore and assess the overall quality of potential nest sites on the basis of several factors. Among others, scouts appear able to evaluate the cleanliness of potential sites, as sites with corpses are avoided, presumably due to disease risk [5].

The presence of deadly pathogens in the selected nest might represent a particularly high cost for social insect colonies. One of the drawbacks of living in dense groups of highly related individuals is the increased chance of pathogen spread and disease outbreaks compared to non-social species [6]. Social insects have evolved a battery of collective anti-parasite defences that complement individual immune resistance to reduce pathogen exposure, transmission and infection rates, also known as “social immunity” [7]. Detecting and avoiding pathogens during the nest site selection process may thus represent a first line of defence.

Pathogens may pose an especially severe problem for invasive ant species. Introduced populations often show reduced genetic diversity [8], [9], as well as a unicolonial social structure, characterized by free exchange of individuals among networks of densely occupied nests [10]. Both factors are expected to make invasive ants particularly prone to epidemics [11]. Avoiding pathogens in the first place is thus expected to be an important strategy for these ants.

The pharaoh ant *Monomorium pharaonis* is a successful “tramp” ant [12] which represents a good candidate to test the ability of invasive species to avoid generalist pathogens during nest emigration. Distributed worldwide [13], this unicolonial species usually thrives in human disturbed environments and nests are often established in ephemeral cavities or household items. Nests are repeatedly subjected to physical disturbance and colonies migrate very readily and frequently split to reproduce by budding. Given the high rate of nest relocation in response to physical disturbance reported in this species [14], as well as the particular mode of colony foundation, pharaoh ant colonies likely experience frequent encounters with a broad range of parasites, including widespread, generalist entomopathogenic fungi of the genus *Metarhizium*.

We therefore investigated the nest site preference of pharaoh ants during nest relocation through a series of binary choice tests between three nest types: nests containing nestmates killed by the entomopathogenic generalist fungus *Metarhizium brunneum* (infected nests), nests containing nestmates killed by freezing (uninfected nests), and empty nests. We predicted that empty nests would be preferred, followed by nests with freeze-killed cadavers, and finally nests containing infectious cadavers.

## Materials and Methods

### Housing and maintenance of ant colonies

Four source colonies of *Monomorium pharaonis* from a laboratory stock population with similar genetic background were used to each create 10 experimental colonies, consisting of 100 workers and 50 larvae, for each of the three different nest choice assays (*n* = 120 in total). Source colonies were reared in plastic boxes (238×203 mm and 175 mm high) with Fluon-coated (BioQuip Products, Rancho Dominguez, CA, USA) walls to prevent escape. Colonies were given fresh water ad libitum in glass tubes sealed with cotton wool and fed twice a week with mealworms (*Tenebrio molitor*), almonds and a 1∶3 proteins to carbohydrates ratio artificial diet [15]. Each colony was also provided with multiple tubes wrapped in aluminium foil, serving as nests. All colonies were maintained at constant temperature and humidity (27±1°C, 65% RH, 12∶12 L/D cycle).

### Experimental arena

**Figure 1:**
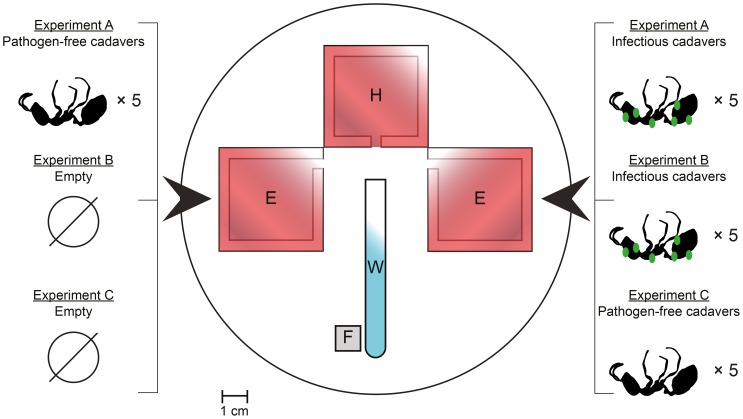
Nest choice assays. In each assay, colonies of *Monomorium pharaonis* were provided with the choice between two experimental nest types: infected vs uninfected nests (experiment A); infected vs empty (experiment B) and uninfected vs empty (experiment C). The position of the experimental nest types was swapped between trials to avoid position biases in the choice. The position of the water tube (W) and food (F) is also showed.

Nest choice assays were performed in experimental arenas consisting of 15 cm diameter Fluon-coated Petri dishes containing a “home” nest and two “experimental” nests (details about their structure can be found in Fig. S1). The three nests in the experimental arena were positioned in order to have the entrances of the two “experimental” nests facing each other and at an equal distance (≈20 mm) from the “home” nest entrance. A water tube was positioned perpendicularly to the entrance of the “home” nest, whereas food (see above) was always located on the bottom-left side of the water tube (see Fig. 1).

### Pathogen infectivity test

The pathogen used in this study was *Metarhizium brunneum* strain ARSEF 1095, formerly named *Metarhizium anisopliae* [16], that came from the USDA-ARS Collection of Entomopathogenic Fungi Cultures in Ithaca, New York, USA and was originally isolated from *Carpocapsa pomonella* [Lepidoptera: Olethreutidae] from Austria. The pathogen was cultured on SDA (Sabouraud Dextrose Agar) for 4 weeks at constant 23°C prior to use. Susceptibility of *M. pharaonis* to the fungal isolate was assessed by dipping individual ant workers (*n* = 42) to conidia suspensions created from sporulating culture plates in a 0.05% solution of a surfactant (Triton X-100, Sigma-Aldrich, St. Louis, USA). The suspensions were previously quantified with a haemocytometer (Neubauer-improved counting chamber) and diluted to the concentration of 2×10^8^ conidia ml^−1^. Before infecting the ants we checked the viability of the conidia by plating 100 µl of 1×10^5^ per ml solution on SDA agar plates, incubating for 19 h at 24°C and recording germination at ×400 magnification (germination rate was 100%).

To confirm that the death of exposed ants was indeed caused by *Metarhizium*, dead ants were surface sterilized following the protocol of Lacey & Brooks [17]. In short, ants were sequentially dipped in 70% ethanol, deionized water (dH_2_O), 5% sodium hypochlorite, and three subsequent changes of dH_2_O. Cadavers were dried on sterile filter paper, and transferred to Petri dishes containing damp filter paper and a wet cotton ball in the centre. Corpses were placed in a circle around the cotton ball, at equal distance from it and from each other. Dead ants were then inspected for fungal growth over 8 days. We also quantified the amount of conidia present on sporulating cadavers by washing them in 1 ml of a 0.05% solution of Triton X. The conidia collected were then counted using a haemocytometer.

The exposure of ant workers to conidia of the fungal isolate resulted in the death of 86% (*n* = 36) of the individuals over 8 days. Three days after the death, 28% of the individuals (*n* = 10) showed mycelia growth on the cuticle. The amount of conidia present on the cuticle was 6.5×10^5^ ml^−1^ (calculated as average of 5 sporulating cadavers).

### Infected cadavers preparation

For the experiment, ant workers were collected from the foraging area of each of the four source colonies and transferred to four Fluon-coated Petri dishes according to their colony of origin. Every Petri dish was provided with the same food used in the experiment, a water tube and several pieces of SDA agar sporulating with the entomopathogenic fungus *M. brunneum* to ensure effective infection.

Every day, dead workers were gently removed from the Petri plates with a soft brush, and surface sterilized as described above. Cadavers were stored at 22±1°C for 2 weeks. Those ants that died from *M. brunneum* infection (confirmed by fungal growth out of the cadaver) and were covered with conidia were gently collected with a brush and transferred into the respective experimental nests.

### Uninfected cadavers preparation

Ant workers were collected from the foraging area of each of the four source colonies and killed by freezing in a −20°C freezer overnight. The following day, cadavers were surface sterilized just as the infected cadavers were, in order to minimize the difference in odour between infected and uninfected cadavers. After the treatment, ant cadavers were transferred to new vials and stored at −20°C. The cadavers were thawed 1 h before being transferred with a brush to the respective experimental nests.

### Nest choice assays

Three types of experimental nests were made: nests containing 5 nestmate cadavers killed by the *M. brunneum* fungus with visible sporulating mycelia (infected nests); nests containing 5 nestmate cadavers killed by freezing (uninfected nests), and empty nests. Cadavers were placed evenly in the nest and at an equal distance from each other. In each nest choice assay, experimental colonies were presented with a choice between two of the experimental nest types. In experiment A, ants were allowed to choose between infected and uninfected nests. In experiment B, the choice was between an empty and an infected nest. In experiment C, ants could choose between an empty and an uninfected nest (Fig. 1).

Assays were prepared 24 h before the start of the experiment by introducing the experimental colony into the home nest of the test arena. The entrances of the two experimental nests were blocked by several layers of Parafilm to avoid exploration by the ants. To avoid bias in the choice because of the asymmetric position of the food in the test arena, the position of the two experimental nests was systematically switched for each replicate. Hence, in each of the three experiments, experimental colonies obtained from the same colony source (*n* = 10) were presented 5 times with a particular experimental nest type on the left and the other 5 times on the right with respect to the home nest entrance.

Nest choice assays were initiated by exposing the home nest to a light source and by the simultaneous removal of the Parafilm blocking the entrance to the experimental nests. Experimental colonies were checked for nest choice (defined as the complete transfer of all the brood items of the experimental colony into one of the two experimental nests) at 10 min intervals within the first hour, and once every hour for the following 4 h.

### Statistical analysis

All statistical analyses were conducted in R [18]. Out of 120 assays, 12 were discarded because ants were able to explore the experimental nests prior to the start of the experiment, and five were discarded because colonies did not move out from the home nest within the 4 h observation period (Table S1). In the remaining 103 assays used for analysis, ants were observed exploring both experimental nests and a nest choice was made within three hours, with 91 (88%) making a choice within the first 30 min (Fig. S2). Once the choice was made, experimental colonies were not found to split between the nests (i.e. all brood items stayed in the nest first chosen).

To test whether the position of the food in the test arena had an influence on the nest choice outcome, for each of the three experiments, we constructed a generalized linear model (GLM) with binomial errors and logit link function, with the type of experimental nest chosen as the dependent variable and the position (left or right) of the nest chosen as a factor, and assessed whether removing the factor “position” from the model had a significant effect based on the likelihood ratio between the two models. Similarly, to test whether the colony of origin of each experimental colony had an effect on the choice outcome, for each of the experiments, we used a likelihood ratio test to compare GLMs with and without colony of origin as an explanatory factor. Since neither the position of the chosen nest nor the origin of the experimental subcolonies had an effect on the choice outcome after correcting for multiple tests, we ran an exact binomial test on the pooled nest choice data for each of the three experiments to test whether there was a preference for a particular experimental nest type. We used a *G*-test to test whether or not the observed responses were consistent across the three experiments.

## Results

Data from replicate colonies were pooled as overall we found no significant colony effect on nest choice in the experiments (likelihood ratio tests between the full model comprising colony origin as factor and the null model comprising only the intercept. Experiment A; infected vs uninfected: χ^2^_3_ = 3.267, *P* = 0.352; experiment B; infected vs empty: χ^2^_3_ = 2.679, *P* = 0.444; experiment C; uninfected vs empty: χ^2^_3_ = 9.397, *P* = 0.024; cutoff *P* = 0.0167 for α = 0.05 when correcting for multiple tests).

**Figure 2:**
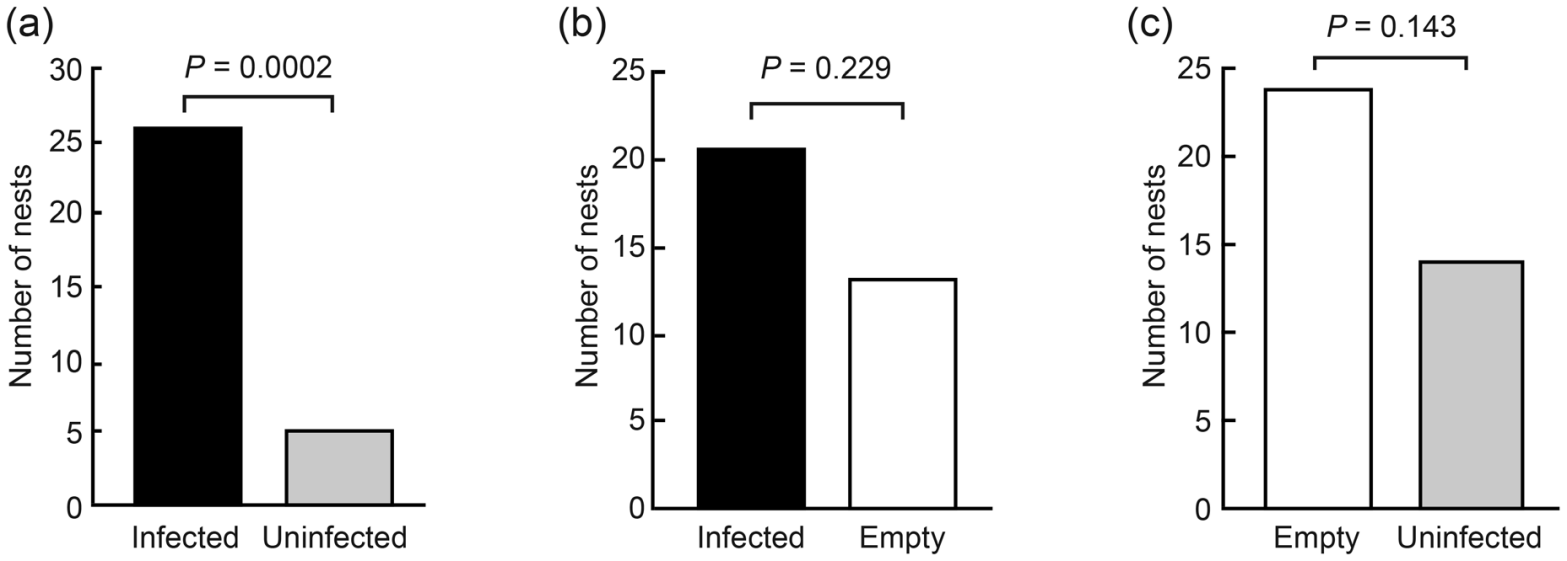
Nest choice of *Monomorium pharaonis* in the three experiments. (a) Infected vs uninfected; (b) Infected vs empty; (c) Uninfected vs empty.

In experiment A, 26 experimental colonies chose infected nests and only five chose uninfected nests, implying that ants had a significant preference for the former (exact binomial test: *P*<0.001, Fig. 2a). In experiment B, 21 experimental colonies chose infected nests and 13 the empty nests (exact binomial test: *P* = 0.229, Fig. 2b). In experiment C, 24 experimental colonies chose the empty nests and 14 uninfected nests (exact binomial test: *P* = 0.143, Fig. 2c).

The position of the experimental nests in the arena did not affect nest choice (likelihood ratio test between the model comprising the position, left or right, of the experimental nest chosen as factor and the null model comprising only the intercept. Experiment A: χ^2^_1_ = 0.169, *P* = 0.681; experiment B: χ^2^_1_ = 1.783, *P* = 0.182; experiment C: χ^2^_1_<0.001, *P* = 1).

The pattern of nest choice across the experiments was similar for the four colonies from which subcolonies were derived (three-way contingency table: *G* ^2^ = 22.06, d.f.  = 17, *P* = 0.182), suggesting that the choices are species rather than colony specific.

## Discussion

We found that emigrating *Monomorium pharaonis* colonies were not only able to discriminate between infected and uninfected nests, but surprisingly, they also displayed a clear preference for the infected nests (Fig. 2). This significant preference for infected nest sites was however not observed when ant colonies could choose between an infected nest and an empty one, although infected nest sites were chosen more frequently. Colonies were also not able to discriminate between uninfected and empty nests, although the latter type was selected more often. All together, our results show that pharaoh ant colonies have a preference for infected nest sites. Empty nests appear to rank as an intermediate choice whereas uninfected nests are the least preferred (Fig. 2). These findings contrast with our initial hypothesis and with studies reporting that insects seem to possess the ability to perceive and avoid direct physical contact with entomopathogenic fungi [19]–[21], possibly using chemicals emitted by fungal spores [22].

One possible explanation of our findings is that either the fungal isolate used or the amount of conidia present on the surface of the sporulating cadavers do not represent a strong lethal threat for the ants, resulting in only a weak repulsive effect. Recent studies showed that workers of the termite species *Macrotermes michaelseni* are not only able to detect the presence of virulent fungi by olfaction, but also that different fungal isolates repelled individuals in dose and virulence related manner [23], [24]. Similar dose-dependent behaviours have been also reported in the ant species *Atta sexdens rubropilosa* [25]. Although we can't completely rule out this hypothesis, preliminary test showed that pharaoh ant workers suffer high mortality after exposure to the *M. brunneum* strain employed in this experiment, confirming its high virulence. Furthermore, even if the amount of conidia present on dead bodies were not representing a genuine threat for the colony, uninfected or empty nests should still represent a better or equal choice when deciding where to relocate the nest given their lower sanitary risk.

The overall preference for infected nest sites brings us to suggest instead that sporulating cadavers may exert an attractive rather than a repulsive effect. A similar effect has been recently reported in the malaria mosquito *Anopheles stephensi*, where female individuals appear to be highly attracted by dead caterpillars infected with the entomopathogenic fungus *Beauveria bassiana* [26]. Likewise, young queens of the ant *Formica selysi* display an initial attraction to nest sites contaminated with entomopathogenic fungi during the dangerous stage of colony foundation [27]. Behavioural responses to sporulating corpses can thus be highly variable between species and the attraction towards fungal conidia does not necessarily need to be seen as non-adaptive. Sporulating cadavers might indeed represent a resource rather than a threat for the host, possibly providing direct or indirect benefits to the individuals coming into contact with. If so, our observed preference order in the choice of the experimental nests might reflect a trade-off process performed by the ants, where infected nests are highly ranked because they provide a valuable resource. Empty nests do not provide benefits or costs, thus ranking as intermediate, whereas nests containing non infected corpses might represent a potential threat and thus least preferred.

One way that infected nests may present an attractive resource is that the ants may use the fungal conidia or the mycelia as a food source, therefore gaining a direct benefit that they would not obtain from the choice of a neutral area such as an empty nest or from a nest containing uninfected corpses. Insect mycophagy is well-known among insects, ranging from the simple ingestion of yeast, mycelium or sporocarps to the elegant symbiosis exhibited by fungal-farming termites and ants [28]. However, the fact that we never observed pharaoh ant workers feeding on cadavers makes this hypothesis unlikely. We argue that it is more plausible that exposure to a fungal pathogen might provide an indirect benefit to the colony.

Recently it was demonstrated that social contact with individuals exposed to fungal pathogens reduces the susceptibility of nestmates to later exposure to the same pathogen [29]. This “social immunization” process requires the transfer of a small number of conidia from exposed to healthy group members by grooming. In turn, healthy individuals pick up low-level inoculum that, although not lethal, leads to an up-regulation of specific immune genes involved in antifungal responses [30]. While this mechanism has so far only been shown in studies involving small groups of workers, individual active immunization could represent an adaptive strategy, leading to the acquisition of colony-level “vaccination” [30]. In order to be beneficial, active immunization of the colony through emigration to infected nest sites requires that the chances of re-encountering the same pathogen are particularly high and the exposure to low pathogen levels through the immunization process confers only a low average risk of mortality to colony members. These conditions appear to be fulfilled in our study system. In fact, the scavenger life-style and the frequent opportunistic nest relocation of pharaoh ants [14] make them likely to have repeated encounters with a common pathogen like *Metarhizium* during their colony life-span. Moreover, the number of conidia present on five nestmate cadavers is unlikely to be sufficient to trigger a deadly disease outbreak in the colony.

The emigration to an infected nest would also increase the possibility that all colony members have access to the pathogen and that they have equal opportunities to prime their immune system. This early vaccination might benefit not only adult individuals, but also larval stages [31]. In contrast, if colonies were only exposed to conidia brought back by scouts, the limited number of conidia together with the compartmentalized social structure of ant colonies [32] would result in limited immunization of the colony. Indeed, previous authors have argued that compartmentalization in the social network of social insects is adaptive precisely because it limits the rapid spread of pathogens from incoming foragers to the rest of the colony [33]. Variation in pathogen mode of transmission and virulence presumably have resulted in different evolved responses, in some cases involving controlled exposure and others complete avoidance. Thus, we suggest that our results are best explained by a social immunization process, although further research is necessary to identify the precise mechanism through which this process can occur.

The observed preference for infected nests over nests containing non infected corpses could also be explained by the higher average risk represented by more virulent undetected pathogens. Since cadavers killed by undetectable pathogens like viral diseases do not readily advertise their cause of death, the choice of infected nests could be interpreted as an active decision of the colony driven by the likely higher threat posed by unknown diseases. That is, it is better to live with a known threat that can be dealt with rather than an unknown threat.

A final hypothesis that could explain our results is that the pathogen attracts its host in order to increase its transmission rate. Examples of pathogen manipulation of host behaviours have been recorded in many species [34]. However most cases involve specialist coevolved parasites, whereas the pathogen we used is a generalist fungal pathogen that targets a broad spectrum of insect hosts.

In conclusion, we showed surprisingly that colonies of the pharaoh ant *Monomorium pharaonis* have an overall strong preference for moving into nest sites infected by the entomopathogenic fungus *Metarhizium brunneum*. Contrary to the current view, we propose that controlled exposure to a low concentration of a common pathogen can provide a long-term benefit for the colony. As already shown in other species, ants can acquire long-term protection against diseases through social contact with previously exposed individuals. This “social immunization” process can in theory lead to colony-wide “vaccination” if all members come into contact with a low level of a particular pathogen. In this respect, our study is the first to show that the need for immunity can lead an entire, functional colony to prefer an infected nest site. Alternatively, the observed preference is non-adaptive and stems from the fungus being able to manipulate the ant host by yet unknown mechanisms. In either case this behavior raises interesting perspectives for biological control, not only of this but also of the many similar ant species that are regarded as some of the world's most problematic invasive pests.

## Supporting Information

Figure S1 **Structure of the artificial nests.** Each nest consisted of four layers: two 40×40 mm glass plates (layer 1 and 3) sandwiching a frame of hard plastic strips (layer 2; width  = 4 mm; height  = 1.5 mm) fixed to the bottom plate with double-sided tape. The top glass plate was covered with a red acetate sheet of equal size (layer 4). Home and experimental nests only differed in the position of the 4 mm wide entrance hole. Floor area was 1024 mm^2^; nest cavity volume was 1536 mm^3^.
(TIF)

Figure S2 **Time needed for experimental colonies to make a choice between the nests offered.** Step plot showing the cumulative time needed by experimental colonies to make a choice, grouped by experiment and based on the pooled dataset (Experiment A: *n* = 31; Experiment B: *n* = 34; Experiment C: *n* = 38).
(TIF)

Table S1 **Data collected during the observation period for each of the three experiments.** *Colony ID* is the original colony from which experimental colonies were created; *Discarded* indicates those assays not used in the analysis because experimental nests were explored before the behavioral observation started or colonies did not move out from the home nest during the observation period (5 h); *# workers* indicates the total number of workers entering experimental nests, including multiple entrances by the same ant, before a choice was made. This data was collected in 5-minute observations during the assay. *Secondary relocation* indicates whether the colony moved first into one experimental nest and then into the other during the observation period. If yes, the last choice was counted as the final decision. *Final choice* is the experimental nest where the colony was located at the end of the observation period. E: empty nest; I: infected nest; U: uninfected nest; H: home nest. *Time* is the period between the start of the experiment and the final choice made by the colony (NA  =  Not applicable).
(DOCX)
