# Supplementary figures and images for: Ant Colonies Prefer Infected over Uninfected Nest Sites
Source: PLoS One. 2014 Nov 5;9(11):e111961. doi: 10.1371/journal.pone.0111961 (PMC4221154; doi:10.1371/journal.pone.0111961)

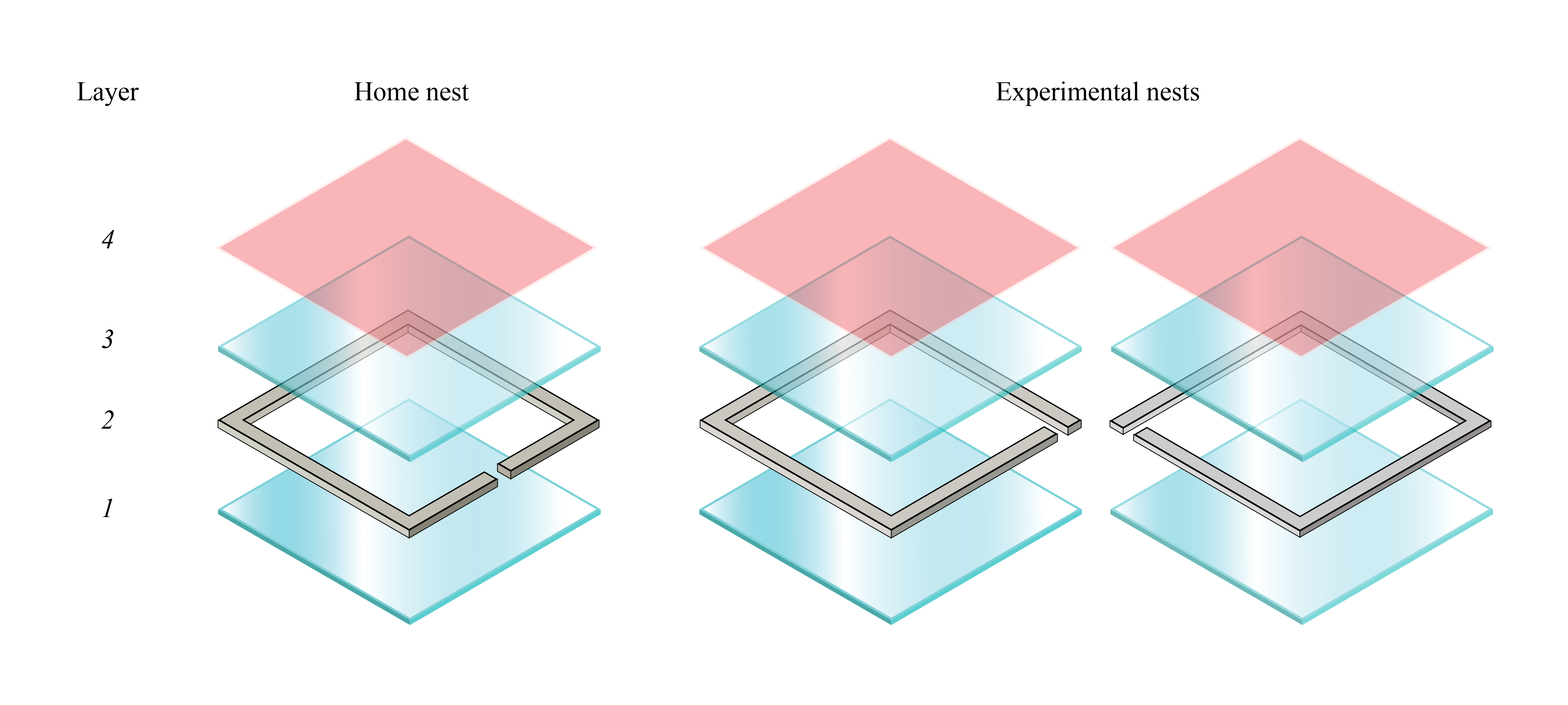

Supplement: Figure S1 — Structure of the artificial nests. Each nest consisted of four layers: two 40×40 mm glass plates (layer 1 and 3) sandwiching a frame of hard plastic strips (layer 2; width = 4 mm; height = 1.5 mm) fixed to the bottom plate with double-sided tape. The top glass plate was covered with a red acetate sheet of equal size (layer 4). Home and experimental nests only differed in the position of the 4 mm wide entrance hole. Floor area was 1024 mm2; nest cavity volume was 1536 mm3. (TIF) [file pone.0111961.s001.tif]

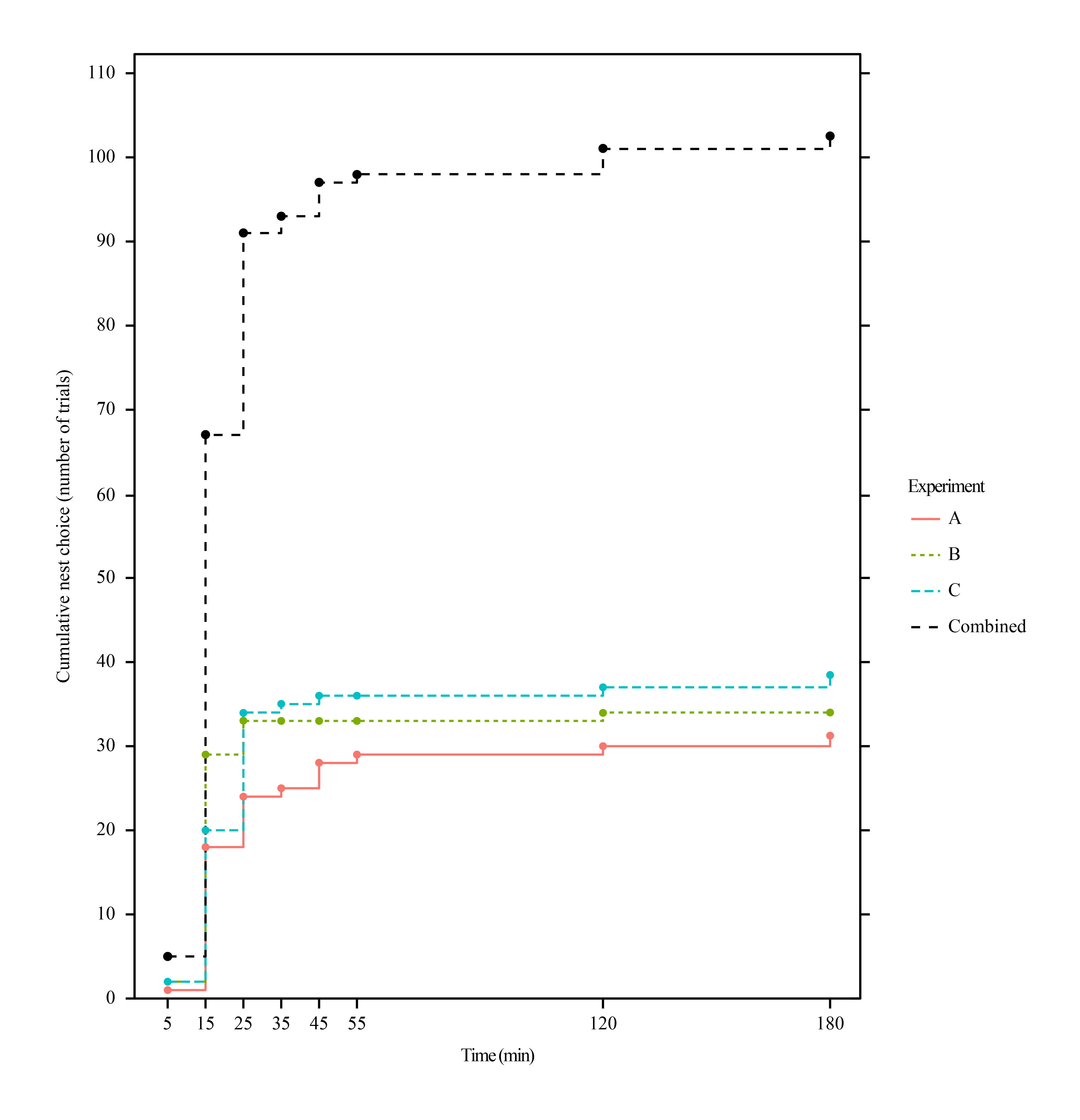

Supplement: Figure S2 — Time needed for experimental colonies to make a choice between the nests offered. Step plot showing the cumulative time needed by experimental colonies to make a choice, grouped by experiment and based on the pooled dataset (Experiment A: n = 31; Experiment B: n = 34; Experiment C: n = 38). (TIF) [file pone.0111961.s002.tif]
