# Supplementary material for: Ant Colonies Prefer Infected over Uninfected Nest Sites
Source: PLoS One. 2014 Nov 5;9(11):e111961. doi: 10.1371/journal.pone.0111961 (PMC4221154; doi:10.1371/journal.pone.0111961)
Supplement: Table S1 — Data collected during the observation period for each of the three experiments. Colony ID is the original colony from which experimental colonies were created; Discarded indicates those assays not used in the analysis because experimental nests were explored before the behavioral observation started or colonies did not move out from the home nest during the observation period (5 h); # workers indicates the total number of workers entering experimental nests, including multiple entrances by the same ant, before a choice was made. This data was collected in 5-minute observations during the assay. Secondary relocation indicates whether the colony moved first into one experimental nest and then into the other during the observation period. If yes, the last choice was counted as the final decision. Final choice is the experimental nest where the colony was located at the end of the observation period. E: empty nest; I: infected nest; U: uninfected nest; H: home nest. Time is the period between the start of the experiment and the final choice made by the colony (NA = Not applicable). (DOCX) [file pone.0111961.s003.docx]

**Table S1 - Experiment A**

| Colony ID | Assay | Discarded | # workers infected | # workers uninfected | Secondary relocation | Final choice | Time |
| --- | --- | --- | --- | --- | --- | --- | --- |
| 4077 | 1 |  | 22 | 5 |  | I | 15 |
| 4077 | 2 |  | 69 | 46 |  | I | 60 |
| 4077 | 3 |  | 11 | 4 |  | I | 25 |
| 4077 | 4 |  | 31 | 28 | yes | U | 60 |
| 4077 | 5 |  | 11 | 10 |  | U | 15 |
| 4077 | 6 |  | 0 | 9 |  | U | 15 |
| 4077 | 7 |  | 2 | 5 |  | I | 15 |
| 4077 | 8 |  | 53 | 21 |  | I | 35 |
| 4077 | 9 |  | 44 | 50 |  | I | 60 |
| 4077 | 10 |  | 2 | 0 |  | I | 15 |
| X2 | 1 |  | 57 | 2 |  | I | 15 |
| X2 | 2 |  | 11 | 75 |  | U | 25 |
| X2 | 3 |  | 102 | 1 |  | I | 5 |
| X2 | 4 |  | 4 | 4 | yes | I | 15 |
| X2 | 5 |  | 57 | 5 |  | I | 15 |
| X2 | 6 |  | 9 | 10 |  | I | 15 |
| X2 | 7 | yes |  |  |  |  |  |
| X2 | 8 | yes |  |  |  |  |  |
| X2 | 9 | yes |  |  |  |  |  |
| X2 | 10 | yes | 56 | 9 |  | H | NA |
| X7 | 1 |  | 39 | 1 |  | I | 15 |
| X7 | 2 | yes | 61 | 11 |  | H | NA |
| X7 | 3 |  | 94 | 41 |  | I | 120 |
| X7 | 4 |  | 117 | 41 |  | I | 60 |
| X7 | 5 | yes |  |  |  |  |  |
| X7 | 6 | yes |  |  |  |  |  |
| X7 | 7 | yes |  |  |  |  |  |
| X7 | 8 |  | 3 | 0 |  | I | 15 |
| X7 | 9 | yes |  |  |  |  |  |
| X7 | 10 |  | 54 | 1 |  | I | 15 |
| X8 | 1 |  | 11 | 2 |  | I | 25 |
| X8 | 2 |  | 1 | 2 |  | I | 15 |
| X8 | 3 |  | 2 | 2 |  | U | 15 |
| X8 | 4 |  | 35 | 1 |  | I | 15 |
| X8 | 5 |  | 4 | 18 |  | I | 15 |
| X8 | 6 |  | 35 | 2 |  | I | 25 |
| X8 | 7 |  | 4 | 0 |  | I | 180 |
| X8 | 8 |  | 0 | 2 |  | I | 25 |
| X8 | 9 |  | 16 | 12 |  | I | 25 |
| X8 | 10 |  | 21 | 64 |  | I | 15 |

**Table S1 (continued) - Experiment B**

| Colony ID | Assay | Discarded | # workers infected | # workers empty | Secondary relocation | Final choice | Time |
| --- | --- | --- | --- | --- | --- | --- | --- |
| 4077 | 1 |  | 6 | 5 |  | E | 15 |
| 4077 | 2 |  | 6 | 6 |  | E | 15 |
| 4077 | 3 |  | 6 | 51 |  | E | 15 |
| 4077 | 4 |  | 4 | 2 |  | E | 15 |
| 4077 | 5 |  | 0 | 8 |  | E | 15 |
| 4077 | 6 |  | 61 | 3 |  | I | 15 |
| 4077 | 7 |  | 44 | 3 |  | I | 15 |
| 4077 | 8 |  | 36 | 11 |  | I | 25 |
| 4077 | 9 |  | 69 | 12 |  | I | 15 |
| 4077 | 10 |  | 60 | 172 | yes | I | 120 |
| X2 | 1 |  | 65 | 3 |  | I | 15 |
| X2 | 2 |  | 1 | 8 |  | I | 15 |
| X2 | 3 |  | 47 | 7 |  | I | 15 |
| X2 | 4 | yes | 61 | 53 |  | H | NA |
| X2 | 5 |  | 39 | 0 |  | I | 15 |
| X2 | 6 |  | 16 | 1 |  | I | 15 |
| X2 | 7 |  | 95 | 3 |  | I | 15 |
| X2 | 8 |  | 84 | 89 | yes | E | 25 |
| X2 | 9 | yes |  |  |  |  |  |
| X2 | 10 | yes |  |  |  |  |  |
| X7 | 1 |  | 55 | 4 |  | I | 15 |
| X7 | 2 |  | 6 | 3 |  | I | 15 |
| X7 | 3 |  | 31 | 2 |  | I | 15 |
| X7 | 4 |  | 2 | 95 |  | E | 15 |
| X7 | 5 |  | 0 | 37 |  | E | 15 |
| X7 | 6 |  | 107 | 6 |  | I | 15 |
| X7 | 7 |  | 87 | 11 |  | I | 15 |
| X7 | 8 | yes | 48 | 87 |  | H | NA |
| X7 | 9 |  | 32 | 104 |  | E | 15 |
| X7 | 10 | yes |  |  |  |  |  |
| X8 | 1 |  | 0 | 141 |  | E | 5 |
| X8 | 2 |  | 0 | 3 |  | E | 15 |
| X8 | 3 |  | 106 | 72 |  | I | 15 |
| X8 | 4 |  | 2 | 0 |  | I | 15 |
| X8 | 5 |  | 19 | 5 |  | I | 15 |
| X8 | 6 |  | 5 | 43 |  | E | 15 |
| X8 | 7 |  | 138 | 48 |  | I | 25 |
| X8 | 8 |  | 8 | 82 |  | E | 25 |
| X8 | 9 |  | 182 | 5 |  | I | 5 |
| X8 | 10 | yes |  |  |  |  |  |

**Table S1 (continued) - Experiment C**

| Colony ID | Assay | Discarded | # workers uninfected | # workers empty | Secondary relocation | Final choice | Time |
| --- | --- | --- | --- | --- | --- | --- | --- |
| 4077 | 1 |  | 18 | 4 |  | U | 15 |
| 4077 | 2 |  | 52 | 22 |  | U | 25 |
| 4077 | 3 |  | 8 | 6 |  | E | 15 |
| 4077 | 4 |  | 25 | 3 |  | U | 15 |
| 4077 | 5 |  | 40 | 61 |  | E | 25 |
| 4077 | 6 |  | 151 | 38 |  | U | 180 |
| 4077 | 7 |  | 125 | 15 |  | U | 25 |
| 4077 | 8 |  | 144 | 13 |  | U | 25 |
| 4077 | 9 |  | 32 | 104 |  | E | 25 |
| 4077 | 10 |  | 57 | 12 |  | U | 25 |
| X2 | 1 |  | 57 | 49 |  | E | 60 |
| X2 | 2 |  | 2 | 11 |  | E | 15 |
| X2 | 3 |  | 5 | 35 |  | E | 15 |
| X2 | 4 |  | 45 | 62 |  | E | 15 |
| X2 | 5 |  | 12 | 92 |  | E | 5 |
| X2 | 6 |  | 6 | 45 |  | E | 25 |
| X2 | 7 |  | 8 | 157 |  | E | 25 |
| X2 | 8 |  | 143 | 31 |  | U | 35 |
| X2 | 9 |  | 21 | 181 |  | E | 15 |
| X2 | 10 |  | 14 | 141 |  | E | 25 |
| X7 | 1 |  | 26 | 25 |  | E | 15 |
| X7 | 2 |  | 48 | 31 |  | E | 120 |
| X7 | 3 |  | 11 | 97 |  | E | 5 |
| X7 | 4 |  | 15 | 7 |  | E | 15 |
| X7 | 5 |  | 32 | 37 |  | E | 25 |
| X7 | 6 |  | 36 | 17 |  | U | 25 |
| X7 | 7 |  | 1 | 52 |  | E | 15 |
| X7 | 8 |  | 27 | 32 |  | H | NA |
| X7 | 9 |  | 10 | 32 |  | E | 15 |
| X7 | 10 |  | 76 | 19 |  | U | 15 |
| X8 | 1 |  | 8 | 7 |  | E | 15 |
| X8 | 2 |  | 19 | 51 |  | E | 25 |
| X8 | 3 |  | 14 | 44 |  | E | 15 |
| X8 | 4 |  | 6 | 9 |  | E | 15 |
| X8 | 5 |  | 51 | 10 |  | U | 15 |
| X8 | 6 |  | 87 | 14 |  | U | 15 |
| X8 | 7 |  | 93 | 8 |  | U | 25 |
| X8 | 8 |  | 158 | 23 |  | U | 25 |
| X8 | 9 |  | 8 | 68 |  | E | 15 |
| X8 | 10 | yes |  |  |  |  |  |
